# The anticonvulsant retigabine suppresses neuronal K_V_2-mediated currents

**DOI:** 10.1038/srep35080

**Published:** 2016-10-13

**Authors:** Jeroen I. Stas, Elke Bocksteins, Camilla S. Jensen, Nicole Schmitt, Dirk J. Snyders

**Affiliations:** 1 Laboratory for Molecular Biophysics, Physiology and Pharmacology, Department of Biomedical Sciences, University of Antwerp, CDE, Universiteitsplein 1, 2610 Antwerp, Belgium; 2 Ion Channel Group, Department of Biomedical Sciences, University of Copenhagen, Blegdamsvej 3, DK-2200 Copenhagen N, Denmark

## Abstract

Enhancement of neuronal M-currents, generated through K_V_7.2-K_V_7.5 channels, has gained much interest for its potential in developing treatments for hyperexcitability-related disorders such as epilepsy. Retigabine, a K_V_7 channel opener, has proven to be an effective anticonvulsant and has recently also gained attention due to its neuroprotective properties. In the present study, we found that the auxiliary KCNE2 subunit reduced the K_V_7.2-K_V_7.3 retigabine sensitivity approximately 5-fold. In addition, using both mammalian expression systems and cultured hippocampal neurons we determined that low μM retigabine concentrations had ‘off-target’ effects on K_V_2.1 channels which have recently been implicated in apoptosis. Clinical retigabine concentrations (0.3–3 μM) inhibited K_V_2.1 channel function upon prolonged exposure. The suppression of the K_V_2.1 conductance was only partially reversible. Our results identified K_V_2.1 as a new molecular target for retigabine, thus giving a potential explanation for retigabine’s neuroprotective properties.

Epilepsy is a complex, debilitating neurological disorder affecting ~1% of the world’s population. Currently, management of epileptic seizures consists of pharmacotherapy; however, in ~20–30% of patients, seizure control cannot be achieved with conventional treatment strategies^1^,^2^. In addition, in many cases drug-resistant epilepsy develops upon prolonged use of antiepileptic drugs (AED’s). These drawbacks have fueled the search for non-conventional treatments and the development of more efficient pharmacotherapy in patients with refractory epilepsy^3^,^4^,^5^.

Retigabine (RTG) is a novel, ‘first-in-class’ anticonvulsant drug approved for use in partial-onset seizures^6^,^7^. Unlike classical AED’s that mainly affect voltage-gated Na^+^ (Na_V_) channels or NMDA/GABA-neurotransmission, retigabine primarily targets voltage-gated K^+^ channels^1^,^2^. Retigabine selectively enhances the low treshold, noninactivating neuronal M-current that regulates spike frequency adaptation and repetitive firing^6^,^8^,^9^. The molecular components of the M-current are the K_V_7.2-K_V_7.5 subunits encoded by *KCNQ2–5* genes respectively. Accordingly, many mutations in the *KCNQ2* and *KCNQ3* genes give rise to distinct epileptic phenotypes further underlining the significance of M-current’s in regulating neuronal excitability^10^,^11^,^12^,^13^,^14^,^15^. Retigabine activates K_V_7 channels by interfering with the normal gating behavior, i.e. retigabine shifts the voltage-dependence of activation to hyperpolarized potentials^16^. As a consequence, K_V_7-mediated currents activate at more negative membrane potentials, effectively hyperpolarizing the resting membrane potential. The binding of retigabine to K_V_7 channels occurs near the pore domain and is dependent on a conserved Trp residue (K_V_7.2 Trp236) that is absent in the K_V_7.1 channel primarily expressed in cardiac and epithelial cells^17^,^18^. More recently, it was found that the binding of retigabine is dependent on the hydrogen-bonding capability of the indole nitrogen atom in the Trp residue and the amide carbonyl oxygen atom of retigabine^19^.

This general mechanism for suppression of neuronal excitability additionally makes retigabine and other K_V_7 activators interesting compounds for several other hyperexcitability-related disorders such as migraine, chronic pain, tinnitus, and even Huntington’s disease^20^,^21^,^22^,^23^. In addition, it has been shown that retigabine has neuroprotective properties^24^,^25^,^26^. However, not all of retigabine’s effects are necessarily due to its action on K_V_7 channels since it also modulates GABA_A_ receptors in a similar concentration range^27^. Due to multiple case reports of long-term toxicity, its clinical application is now restricted to patients for whom other anticonvulsant drugs have proved inadequate^28^,^29^,^30^. Whether this toxicity arises from off-target retigabine receptors or chronic activation of K_V_7 channels remains unknown. Surprisingly, despite the large structural similarities within the K_V_ channel family, little effort has been made to determine whether other K_V_ channels are modulated by retigabine^7^. In addition to this, it has not yet been investigated whether the accessory subunit KCNE2 impacts the retigabine effect on K_V_7 despite the fact that K_V_7.2-K_V_7.3 channels and KCNE2 have overlapping expression patterns and described gating effects^31^,^32^.

Here, we performed an electrophysiological screening on members of the K_V_1–K_V_9 and K_V_11 subfamilies to investigate whether these channels are affected by retigabine. We found that retigabine inhibited all K_V_ channels tested, but that this inhibition only occurred in the high μM range, with the exception of K_V_2.1. Inhibition of K_V_2.1 required only low μM concentrations and was only partially reversible. In addition, we found that the addition of the auxiliary subunit KCNE2 decreased the retigabine sensitivity of heterotetrameric K_V_7.2-K_V_7.3, but not of K_V_2.1, channels. These findings identify K_V_2.1 as an important molecular target for the action of retigabine and, due to K_V_2.1’s key role in apoptosis, could help explain the previously reported neuroprotective properties^24^,^25^,^26^.

## Results

### KCNE2 modulates retigabine sensitivity of K_V_7.2-K_V_7.3 channels

**Figure 1:**
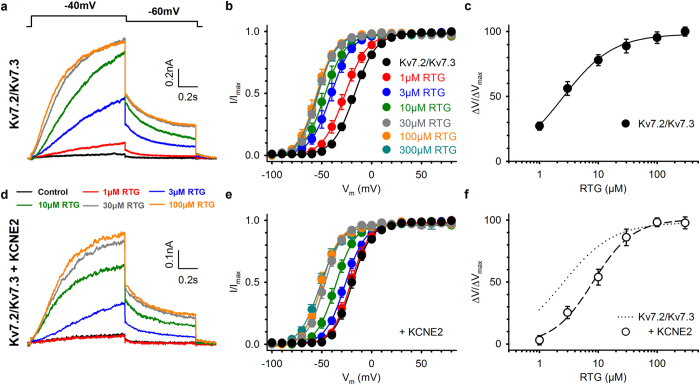
KCNE2 decreases the retigabine sensitivity of heterotetrameric K_V_7.2-K_V_7.3 channels. (**a**) Effect of increasing concentrations of retigabine (1–100 μM) on K_V_7.2-K_V_7.3 currents. Retigabine potentiated the K_V_7.2-K_V_7.3 current in a concentration-dependent manner, and saturation occurred above 30 μM. Voltage protocol is shown on top. (**b**) Voltage-dependence of activation. Increasing concentrations of RTG caused a gradual hyperpolarizing shift. (**c**) Concentration-effect curve plotted as the shift in the voltage-dependence of activation normalized to the maximal observed shift (ΔV/ΔV_max_) as function of the drug concentration. (**d**) Similar to (**a**) but after co-expression with KCNE2. Retigabine potentiated the K_V_7.2-K_V_7.3-KCNE2 currents but unlike (**a**) concentrations above 1 μM had to be used. (**e**) Voltage-dependence of activation. KCNE2 reduced the hyperpolarizing shift at every drug concentration, and decreased the maximal observed shift (ΔV_max_). (**f**) Concentration-effect curve.

The pharmacology of K_V_7 channels is highly dependent on its association with auxiliary KCNE subunits^33^,^34^. Of the five known KCNE proteins, KCNE2 potentially interacts with the main determinants of the M-current - heteromeric K_V_7.2-K_V_7.3 channels - based on overlapping expression patterns and gating effects^31^,^32^. Hence, we co-transfected K_V_7.2-K_V_7.3 with YFP-KCNE2 enabling us to select KCNE2-transfected cells. Retigabine potentiated K_V_7.2-K_V_7.3 currents in a concentration-dependent manner (Fig. 1a) by shifting the voltage-dependence of activation to hyperpolarized potentials (Fig. 1b) with an EC_50_ of 1.9 ± 0.3 μM and a Hill coefficient of 1.4 ± 0.1 (n = 11; Fig. 1c), as previously reported^7^,^16^,^17^. Co-transfection of KCNE2 with the K_V_7.2-K_V_7.3 channel complexes did not prevent retigabine from potentiating the current (Fig. 1d). However, KCNE2 reduced the shift in the voltage-dependence of activation (ΔV) at every concentration (Fig. 1e), resulting in a right-shifted concentration-effect curve with an EC_50_ of 10.0 ± 2.2 μM and a Hill coefficient of 1.0 ± 0.1 (n = 9; Fig. 1f). In addition, the maximal shift in the voltage-dependence of activation (ΔV_max_) was reduced from 39.2 ± 1.4 mV (n = 11) under control conditions to 30.7 ± 1.1 mV (n = 9) in the KCNE2-transfected cells (see Supplementary Table S1). Thus, KCNE2 reduced the retigabine sensitivity of K_V_7.2-K_V_7.3 channels approximately 5-fold.

### Retigabine inhibits K_V_ channels

**Figure 2:**
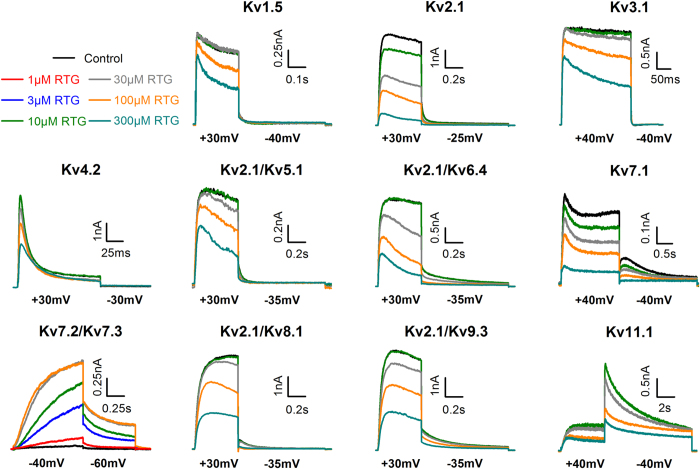
Retigabine inhibits most K_V_ channels in the intermediate to high μM range. A two-step pulse protocol adjusted to the biophysical properties of the respective channel was used. The voltage applied is shown below the respective K_V_ channel current traces. Retigabine (colored traces) inhibited all K_V_ channels in the high μM range (>100 μM) with exception of K_V_2.1, which was inhibited at relative low μM concentrations (10 μM).

To determine whether retigabine affected other Kv channels, we screened representative channels of the K_V_1–K_V_9 and K_V_11 subfamilies (Fig. 2). A two-step screening pulse adjusted to the biophysical properties of the respective channel was used. Since they cannot form homotetrameric channels at the plasma membrane, members of the K_V_5, K_V_6, K_V_8, and K_V_9 subfamilies where co-transfected with K_V_2.1^35^. Retigabine inhibited all K_V_ channels tested, though only at high μM concentrations (>100 μM). However, K_V_2.1 currents were inhibited at relatively low concentrations (10 μM) (Fig. 2). Interestingly, this increased sensitivity was absent when K_V_2.1 co-assembled respectively with K_V_5.1, K_V_6.4, K_V_8.1, and K_V_9.3 subunits.

### K_V_2.1 inhibition is voltage-dependent and only partially reversible

**Figure 3:**
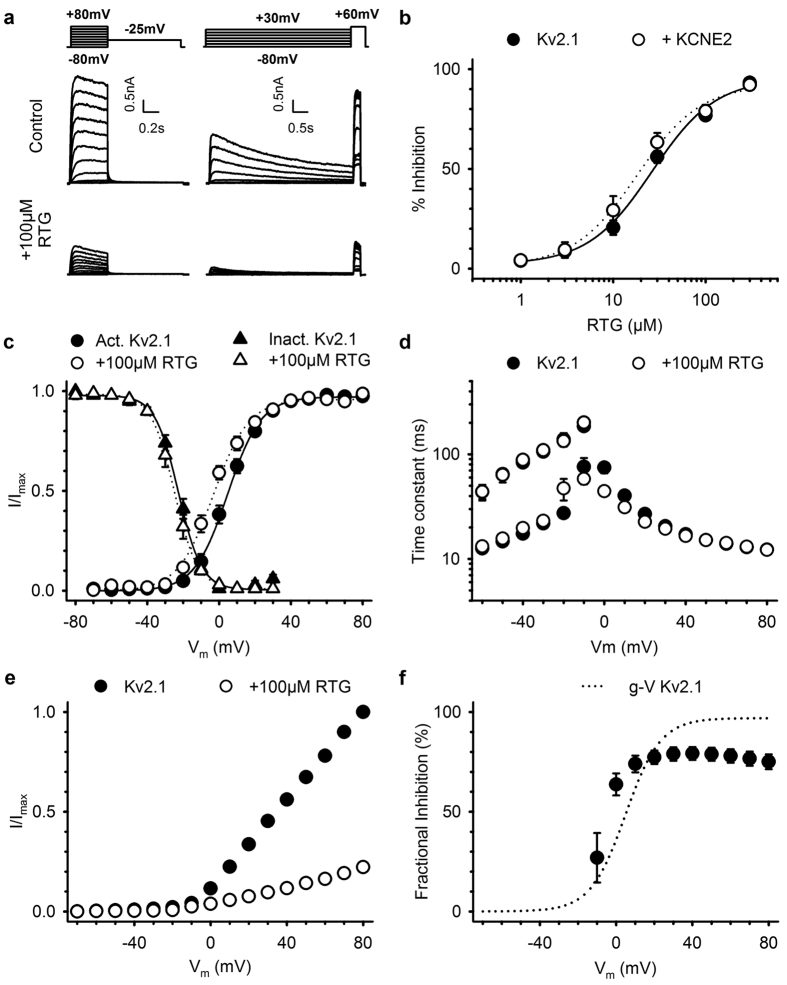
Retigabine inhibition of K_V_2.1 is voltage-dependent. (**a**) Typical current recordings of K_V_2.1 channels to determine the activation (left) and inactivation (right) properties, before (top) and after exposure to 100 μM retigabine (bottom). Voltage protocols are shown on top. (**b**) Concentration-effect relationship of K_V_2.1 inhibition. Retigabine inhibition of K_V_2.1 (closed circles) currents was not significantly different (p = 0.385) in the presence of KCNE2 (open circles). (**c**) Voltage-dependence of activation (circles) and inactivation (triangles) in absence (closed symbols) and presence (open symbols) of 100 μM retigabine. The voltage-dependence of activation was obtained by plotting the normalized tail currents (I/I_max_) in the activation current traces from panel A as function of the prepulse potential. Retigabine induced a small but significant (p = 0.012) hyperpolarizing shift in the voltage-dependence of activation. The voltage-dependence of inactivation, obtained by plotting the normalized peak current (I/I_max_) at +60 mV after a 5 s prepulse as a function of the prepulse potential, was not affected by retigabine. (**d**) Time constants of K_V_2.1 channel opening (≥0 mV) and closing (<0 mV) in absence (filled circles) and presence (open symbols) of 100 μM retigabine. (**e**) Current-voltage (I-V) relationship, obtained by plotting the current at the end of the 500 ms varying pulse as function of the voltage. (**f**) Fractional inhibition as function of the applied voltage. The fractional inhibition, obtained by dividing the I-V relationships in (**e**) displayed significantly less inhibition at weak depolarizing potentials. The dotted line represents the voltage-dependence of activation of K_V_2.1.

To gain insight into the underlying mechanism of K_V_2.1 channel inhibition, we performed a detailed biophysical characterization of retigabine effects. Retigabine inhibited K_V_2.1 currents with an IC_50_ of 22.0 ± 1.6 μM and Hill coefficient of 1.6 ± 0.1 (n = 5; Fig. 3b). Although KCNE2 was previously found to interact with K_V_2.1^33^, it did not alter the IC_50_ for inhibition. Retigabine inhibition of K_V_2.1-KCNE2 currents occurred with an IC_50_ of 16.1 ± 1.8 μM (p = 0.056) and a Hill coefficient of 1.4 ± 0.2 (p = 0.384) (n = 6). No change could be observed in the voltage-dependence of inactivation (triangles, Fig. 3c) or the time constants of channel opening/closing (Fig. 3d). However, retigabine did induce a small but significant hyperpolarizing shift of approximately 6 mV (p = 0.012), from 3.7 ± 1.5 (n = 12) to −2.5 ± 1.6 mV (n = 9), in the voltage-dependence of activation (circles, Fig. 3c and Table 1). Next, we determined the voltage-dependence of channel inhibition (or fractional inhibition), obtained by dividing the current-voltage (I-V) relationships in Fig. 3e, and plotted this alongside the voltage-dependence of activation (Fig. 3f). Inhibition of K_V_2.1 displayed a clear voltage-dependency; less inhibition occurred at weak depolarizing potentials where only a small fraction of K_V_2.1 channels conducted current. 100 μM retigabine inhibited 78.0 ± 3.4% (n = 7) of the current at +60 mV while only 27.0 ± 12.4% (n = 7) inhibition occurred at −10 mV (Fig. 3f).

**Figure 4:**
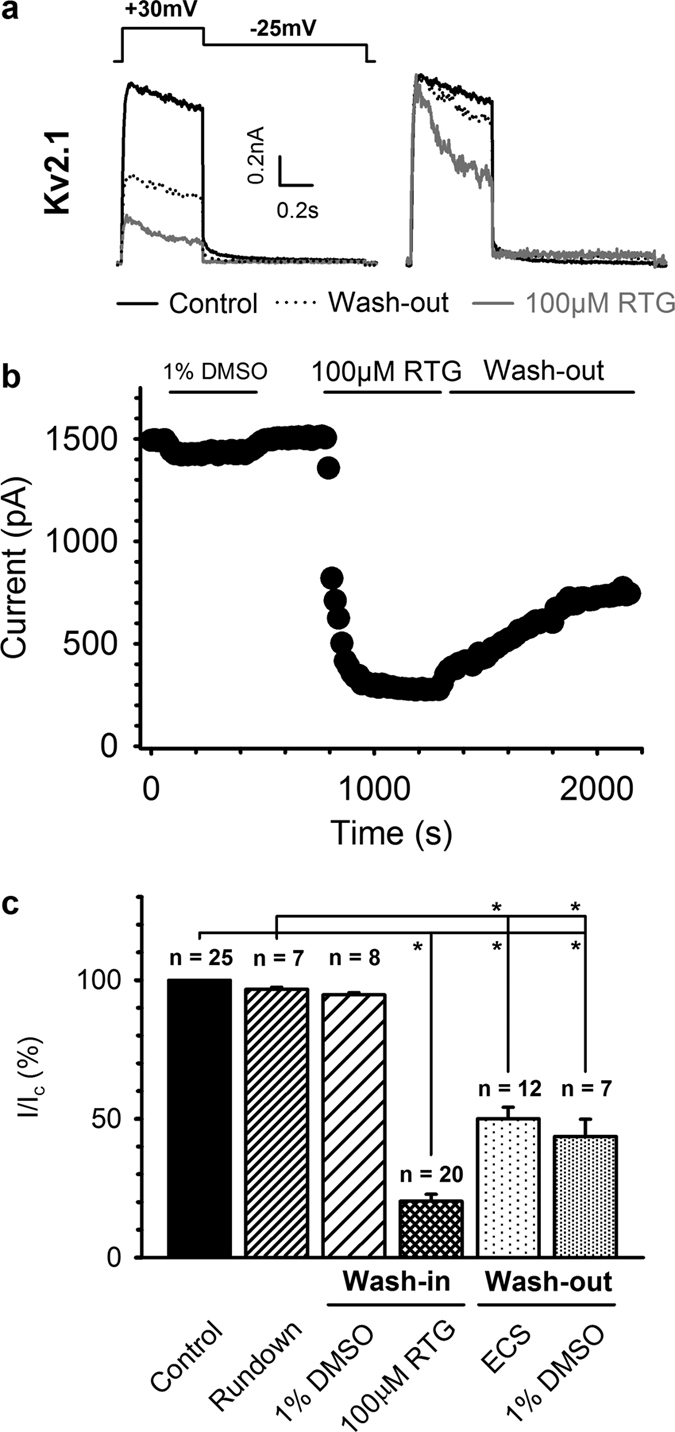
Inhibition of K_V_2.1 current by retigabine is only partly reversible. (**a**) Representative K_V_2.1 current traces (black) at +30 mV (left). The scaled current traces are shown in the right panel. Retigabine (grey) inhibited approximately 80% of the current but inhibition was poorly recovered 30 minutes after removal of retigabine (dotted). The ‘apparent’ acceleration of the inactivation process seen in the scaled current traces most likely reflects open-channel block by retigabine. (**b**) Plot of a representative wash-in/wash-out experiment. Inhibition of the K_V_2.1 current occurred slowly, typically requiring 5–10 minutes to achieve saturation. Inhibition of K_V_2.1 currents was poorly reversible and occurred extremely slow. (**c**) Bar chart illustrating the degree of current (I/I_c_), with I the current at a given condition and I_c_ the control condition. K_V_2.1 inhibition was poorly reversible, independent of the solvent, and significantly different from current rundown. *Indicates statistical significance (p < 0.05).

Furthermore, we determined the wash-in/wash-out kinetics of K_V_2.1 channel inhibition at +30 mV where the voltage-dependence of inhibition was maximal (see Fig. 3f). Inhibition of K_V_2.1 currents was only partially reversible and appeared to accelerate the development of channel inactivation, indicating an open-channel block mechanism (Fig. 4a). Inhibition of K_V_2.1 by 100 μM retigabine was slow, with a τ_wash-in_ of 89.7 ± 14.4 s (n = 10), on average requiring ± 10 minutes before inhibition was saturated (Fig. 4b). Recovery of inhibition was markedly slower, with a τ_wash-out_ of 574 ± 67 s (n = 8), and incomplete with only 41.8 ± 7.6% recovery in 30 minutes (Fig. 4d and Table 1). Recovery of inhibition was significantly different from normal rundown (p < 0.001) and was not dependent on the solvent: addition of 1% DMSO did not increase the rate of recovery (Fig. 4c). Next, we investigated the state-dependence of inhibition to determine whether retigabine was capable of inhibiting K_V_2.1 channels in their closed-state (Supplementary Fig. S1a). Application of retigabine during a 300 s pulse to −90 mV (where all channels are closed) inhibited the peak and ‘end’ current at a test pulse to +30 mV compared to the control (Supplementary Fig. S1a,b). However, subsequent recording of a train of pulses revealed that this degree of inhibition was significantly different from saturated inhibition. Interestingly, during the ‘conventional’ wash-in experiments the inhibition saturated within 300 s (Fig. 4b). Thus, these observations argue against efficient inhibition of closed K_V_2.1 channels by retigabine. Evidence for this inefficient inhibition of closed K_V_2.1 channels was further strengthened when we compared the ‘peak’ and ‘end’ currents of the wash-in protocols illustrated in Fig. 4b (Supplementary Fig. S1c), The ‘peak’ current during the next step was always 10–15% larger than at the end of the previous pulse. This indicates that no significant additional inhibition developed during the 14.5 s interval at −80 mV. Given the similar ‘recovery’ independent of the level of inhibition, this most likely reflects recovery from slow inactivation at −80 mV.

The slow onset of inhibition combined with the incomplete recovery raised the question whether clinically relevant concentrations of retigabine could affect K_V_2.1 when the exposure time was increased. To investigate this, we performed ‘incubation’ experiments (illustrated in Supplementary Fig. S2a). HEK cells were transfected and exposed to low concentrations of retigabine (0.1–3 μM) for 4 hours. Interestingly, retigabine reduced the K_V_2.1 current density in a concentration-dependent manner, independent of the manipulation or solvent (Supplementary Fig. S2b). The current density was significantly reduced by approximately 2.5-fold after exposure to 1 (p = 0.027) and 3 μM (p = 0.024) retigabine (Supplementary Fig. S2c). To exclude the possibility that the reduced current densities occurred as a consequence of altered K_V_2.1 channel gating, we determined the voltage-dependence of activation for each condition, and found that it was not modified (Supplementary Fig. S2d). A full biophysical characterization was performed for control and exposure to 0.1% DMSO and 1 μM retigabine, but no significant changes were observed (data not shown).

### Retigabine inhibits native K_V_2.1 currents in rat hippocampal neurons

**Figure 5:**
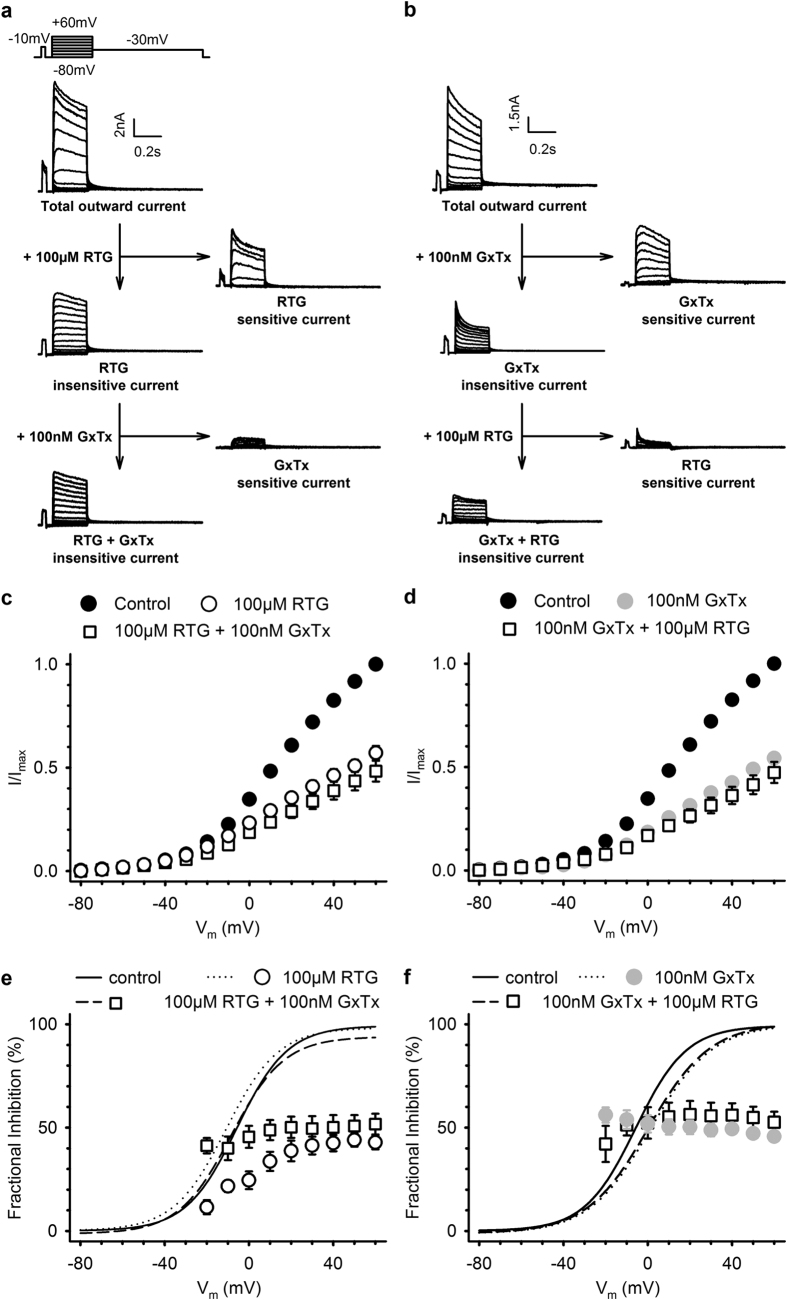
Retigabine inhibits the K_V_2-mediated component of the outward current in cultured rat hippocampal neurons. (**a**) Representative current traces from cultured rat hippocampal neurons. 100 μM retigabine inhibited the outward current and the RTG-sensitive current was obtained after subtraction. 100 nM Guangxitoxin-1E (GxTx), i.e. selective K_V_2 inhibitor, was used to confirm the inhibition of K_V_2-mediated current by retigabine. Retigabine inhibited a major component of delayed rectifier current, with little inhibition caused by GxTx (**b**) similar to (**a**) although the K_V_2-mediated current was first inhibited with GxTx before applying retigabine. Inhibition of the K_V_2-mediated component of the current by GxTx resulted in little inhibition of retigabine. However, retigabine still inhibited a fast activating and inactivating current. (**c,d**) Current-voltage relationship, obtained by plotting the total outward current at the end of the 250 ms step as function of the voltage with retigabine (**c**) or GxTx (**d**) initial exposure. (**e,f**) Fractional inhibition as a function of the applied voltage. As observed in HEK cells (Fig. 3f), retigabine (**e**) had a voltage-dependence of inhibition that could be abolished after subsequent exposure to GxTx. Panel (**f**) is similar to (**e**) but with initial exposure to GxTx. Lines represent the voltage-dependence of activation fitted with the Boltzmann equation.

To determine whether the inhibition observed in an overexpression system translated to similar effects in an *in vivo* setting, we tested native K_V_2-mediated currents in cultured rat hippocampal neurons. Total outward currents were recorded with a prepulse to −10 mV to eliminate most of the I_A_ current, as previously described^36^. 100 μM retigabine was used for this purpose because: 1) it caused significant inhibition of K_V_2-mediated currents and 2) did not substantially inhibit other K_V_ channels (see Fig. 2). The degree of inhibition was determined at the end of the 250 ms pulse. As expected, retigabine caused significant inhibition, 44.0 ± 3.2% at +60 mV (n = 9), of the total outward current in cultured rat hippocampal neurons (Fig. 5a). To further identify the retigabine inhibited currents as K_V_2.1-mediated, we used Guangxitoxin-1E (GxTx-1E), a selective Kv2 inhibitor^37^. We used a concentration of 100 nM GxTx-1E that has been reported to produce near-saturating effects on K_V_2-mediated currents in mice CA1 hippocampal neurons^37^. GxTx-1E caused little additional inhibition (8.2 ± 4.8%, n = 6 and p = 0.146) of the total outward current suggesting that retigabine inhibited the majority of K_V_2-mediated currents. To validate these results, we performed the experiments in the reverse order: K_V_2-mediated currents were first inhibited with GxTx-1E, before retigabine was applied (Fig. 5b). GxTx-1E inhibited 45.7 ± 2.0% (n = 6) of the total outward current at +60 mV and retigabine did not cause significant additional inhibition (6.9 ± 3.6%, n = 6 and p = 0.589). As an additional control, we repeated the experiments with 5 μM tetrodotoxin (TTX) in the bathing solution, in order to block Na_V_ channels (Supplementary Fig. S3). Retigabine still inhibited a major component of the total outward current in the presence of extracellular TTX, with little additional inhibition caused by GxTx-1E (n = 3)(Supplementary Fig. S3a). The normalized current-voltage relationships confirmed these observations. When K_V_2-mediated currents were not first inhibited with GxTx-1E, retigabine inhibited a major component of the total outward current (Fig. 5c,d and Supplementary Fig. S3c). Interestingly, as observed for the retigabine inhibition of K_V_2.1 in HEK cells, retigabine inhibited the total outward current in a voltage-dependent manner with decreased sensitivity at weak depolarizing potentials.

## Discussion

**Figure 6:**
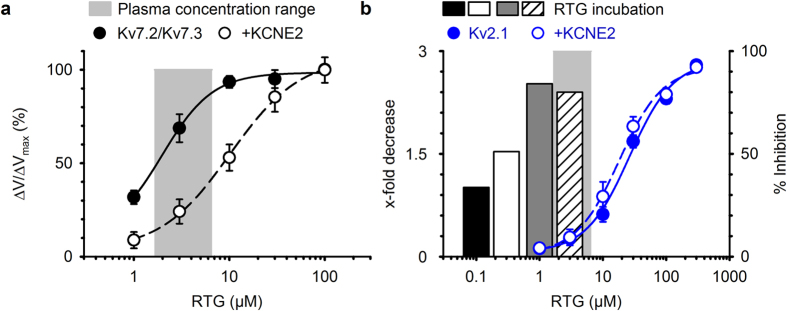
Molecular pharmacology on K_V_2 and K_V_7 channels in HEK cells compared with retigabine plasma concentrations. (**a**) Concentration-effect relationship of retigabine potentiation on K_V_7.2-K_V_7.3 currents in the absence (filled circles) and presence (open circles) of KCNE2. The grey bar represents the plasma concentration range, minimum (0.65 μM) to maximum (6.6 μM), in patients treated with 600–1200 mg retigabine/day^38^,^39^,^40^,^41^,^42^. Although KCNE2 shifted the concentration-effect curve, K_V_7.2-K_V_7.3 current potentiation was not fully prevented in the plasma concentration range. (**b**) Concentration-effect relationship of K_V_2.1 inhibition in absence (blue, filled circles) and presence (blue, open circles) of KCNE2, obtained from direct perfusion of retigabine on the Kv2.1 channels. The light grey bar represents the plasma concentration range as in (**a**). Black, white, dark grey and striped bar represent the x-fold reduction in K_V_2.1 current density obtained from the retigabine incubation experiments. Although little direct K_V_2.1 inhibition occurred, maximal suppression of the K_V_2.1 current density occurred in the plasma concentration range.

Enhancement of K_V_7 channel activity by retigabine provides a general mechanism for suppression of multiple hyperexcitability-related disorders such as epilepsy, chronic pain and tinnitus^20^,^21^,^22^. Our results illustrate that the auxiliary KCNE2 subunits reduced the retigabine sensitivity of K_V_7.2-K_V_7.3 by approximately 5-fold. Although the role of KCNE2 in the nervous system and its interaction with K_V_7.2-K_V_7.3 channels remain controversial, the potential of KCNE2 to modulate K_V_ channel pharmacology is well established^31^,^33^. The interaction between KCNE2 and K_V_7.2-K_V_7.3 channels alters the biophysical properties modestly^32^. Our study showed that KCNE2 reduced the retigabine sensitivity of K_V_7.2-K_V_7.3 channels, further supporting the idea that KCNE2 can interact with these K_V_7.2-K_V_7.3 channels. However, we did not observe the previously reported KCNE2-induced changes in the biophysical properties. In our experiments, KCNE2 had the tendency to hyperpolarize the voltage-dependence of activation. Even though KCNE2 reduced the retigabine sensitivity of K_V_7.2-K_V_7.3 channels, it still had some effect within the clinical plasma concentration range as illustrated in Fig. 6a where we show the concentration-effect curves together with the retigabine plasma concentration range^6^,^38^,^39^,^40^,^41^,^42^.

By demonstrating that clinically relevant retigabine concentrations inhibited K_V_2-currents, presumably through an open channel block mechanism, both in HEK cells and hippocampal neurons (Fig. 6b), our findings indicate that K_V_2 channels might represent an important ‘off-target’ receptor responsible for some of retigabine’s (adverse) effects. Although acute exposure of K_V_2.1 channels to retigabine resulted in inhibition at concentrations above the clinical range (blue circles Fig. 6b), the prolonged incubation experiments (bars Fig. 6b) revealed a strong reduction of the K_V_2.1 current density at clinical, and even sub-clinical concentrations of retigabine. As a result, retigabine might exert a significant effect on K_V_2.1 channels *in vivo*. Pharmacological suppression of K_V_2 channels results in either an increase or decrease of neuronal excitability, as previously shown with the K_V_2-selective inhibitor GxTx-1E^37^. GxTx-1E inhibits between 60–80% of the total delayed rectifier current in rat superior cervical ganglion neurons and mouse hippocampal CA1 neurons. This results in an increased initial firing frequency but depresses maintained firing. 100 nM GxTx-1E only inhibited ~50–55% of total outward current in our experiments. This discrepancy most likely arises from differences in the age of the hippocampal neurons used: Liu and Bean performed recordings on acutely dissociated hippocampal neurons while our recordings were performed on hippocampal neurons that were cultured for 10–15 days *in vitro*.

K_V_2.1 channel expression is rather ubiquitous and serves major physiological functions in the central nervous system and (neuro)endocrine cells^43^,^44^. K_V_2.1 channels constitute the major delayed rectifier current in hippocampal neurons, and targeted deletion of K_V_2.1 results in neuronal and behavioral hyperexcitability^37^,^45^,^46^. The expression pattern of K_V_2.1 channels is intriguing, in that they form cell-surface clusters at the soma, proximal dendrites and the axon initial segment, not only in cultured hippocampal neurons and intact brain but also in transfected HEK cells^47^,^48^. Interestingly, within the clusters, K_V_2.1 channels are inactive, i.e. gating charge movement of the voltage sensing domains was detected without measurable ionic currents, and upon dispersal, the ionic current is regained. Within the micro-domain of the cluster, K_V_2.1 channels are in close proximity to the endoplasmatic reticulum (ER) and induce the formation of ER-plasmamembrane junctions^49^,^50^,^51^. Thus, it was suggested that K_V_2.1 cell surface clusters are insertion platforms for ER-membrane trafficking^52^. Clustering of K_V_2.1 is highly dependent on the phosphorylation state of the channel and directly linked to underlying neuronal activity. This means that hyperexcitability, as observed during epileptic seizures, promotes dephosphorylation of K_V_2.1 through a Ca^2+^/calcineurin-dependent mechanism which leads to increased K_V_2.1 activity and K_V_2.1 declustering^36^,^53^,^54^. Interestingly, K_V_2.1 clusters can also be dispersed pharmacologically. When hippocampal neurons are exposed to glutamate, rapid K_V_2.1 declustering occurs^55^. Due to the fact that retigabine inhibited K_V_2.1 currents in a poorly reversible manner, both in HEK cells and hippocampal neurons, K_V_2.1 trafficking might be affected upon retigabine exposure. One could speculate that loss of functional K_V_2.1 channels at the cell surface, due to increased endocytosis or changed clustering pattern, might contribute to retigabine’s poorly reversible inhibition of Kv2.1 currents. However, using live-cell imaging of GFP-tagged K_V_2.1, we did not observe a change in the K_V_2.1 localization within 30 min after exposure to retigabine (data not shown). Interestingly, K_V_2.1 current densities were reduced with much lower retigabine concentrations (0.1–3 μM) upon prolonged exposure. In this case, the reduced K_V_2.1 current density might simply reflect the population of K_V_2.1 channels that were not inhibited by retigabine as the gating and trafficking (data not shown) properties were not modified. In addition, it cannot be excluded that the observed reduction in K_V_2.1 current density is (partially) caused by a drug metabolite of retigabine. On the other hand, retigabine’s hydrophobic nature suggests that it might reside in the lipid bilayer or bind to a hydrophobic region in the K_V_2.1 channel. Although this could help explain the poor reversibility of K_V_2.1 current inhibition upon wash-out, it seems rather unlikely, because: 1) increased solvent concentrations had no significant effect on the reversibility of inhibition (Fig. 4c), and 2) retigabine’s action on K_V_7 channels was always fully reversible. An interesting future direction will be to further investigate the underlying mechanism of this poorly reversible reduction in K_V_2.1 current densities.

Interestingly, K_V_2.1 might be involved in the neuroprotective properties of retigabine that have been described more recently, especially due to the key role of K_V_2.1 in apoptosis^56^,^57^. Upon neuronal injury, K_V_2.1-currents are increased through *de novo* insertion of channels in the plasma membrane and subsequent decrease of intracellular [K^+^] levels promoting activation of the apoptotic cascade^58^. The extent of cell death can be reduced when the pro-apoptotic K_V_2.1 current is pharmacologically inhibited^59^. Therefore, retigabine might prevent apoptosis through inhibition of K_V_2.1 currents, thus promoting cell survival. However, retigabine was found to promote neuroprotection by diminishing excitotoxicity through suppression of hyperexcitability by K_V_7 channel activation^24^,^25^,^26^. Thus, retigabine might promote neuroprotection in neurons through its concerted action on K_V_2 and K_V_7 channels.

In conclusion, we found that the retigabine sensitivity of K_V_7 channels is reduced by the auxiliary KCNE2 subunit. In addition, retigabine inhibited K_V_2.1 channels most likely through an open-channel block mechanism in a poorly reversible manner at clinically relevant concentrations.

## Materials and Methods

### Molecular biology

Human K_V_1.5 (GenBank Accession Number NM_002234), K_V_2.1 (NM_004975), K_V_3.1 (NM_004976), K_V_4.2 (NM_012281), K_V_5.1 (NM_002236), K_V_6.4 (NM_172347), K_V_8.1 (NM_014379) and K_V_11.1 (NM_000238), as well as mouse K_V_9.3 (NM_173417), were subcloned in the eGFP-N1 vector (Clontech, Palo Alto, CA, USA). Human K_V_7.1 (NM_000218), K_V_7.2 (NM_172107), K_V_7.3 (NM_004519), KCNE2 (NM_172201) and YFP-KCNE2 were subcloned in the pBK/CMV vector as described previously^60^.

### Transient transfection and cell culture

HEK293 cells were cultured in 60 mm cell culture dishes filled with 4 ml culture medium - consisting of Dulbecco’s modified Eagle’s medium supplemented with 10% horse serum, 1% penicillin/streptomycin and 1% non-essential amino acids–under physiological conditions (37 °C and 5% CO_2_). HEK293 cells were transiently transfected with 0.05–5 μg cDNA of the respective channel together with the GFP transfection marker using Lipofectamine2000 (Invitrogen, San Diego, CA, USA), according to the manufacturer’s instructions. To obtain the characteristic K_V_7.2-K_V_7.3 currents both K_V_ subunits were co-transfected in a 1:1 molar ratio. Co-transfection with KCNE2 was performed in 1:1:4 and 1:4 molar ratios for K_V_7.2-K_V_7.3 and K_V_2.1 channels, respectively. The amount of cDNA was kept identical between the–KCNE2 and +KCNE2 experiments by addition of empty vector cDNA. A C-terminal YFP-KCNE2 construct was used to allow for selection of KCNE2-transfected cells. The YFP-KCNE2 behaved similarly to the untagged KCNE2. 16–24 h after transfection, HEK293 cells were dissociated with trypsine and transferred to the patch-clamp set-up for electrophysiological analysis.

The retigabine incubation experiments were performed on HEK293 cells transfected with 10 ng K_V_2.1 over 48 h. 24 h post-transfection, HEK293 cells were either exposed to normal medium (control) containing 0.1% DMSO (vehicle control) or 0.1, 0.3, 1 or 3 μM retigabine for 4 h. After the 4 h exposure, the incubation medium was removed and fresh medium was added to the transfected HEK293 cells. Thus, at the moment of electrophysiological analysis (48 h post-transfection), no retigabine was present in the recording solution.

### Primary cultures of rat hippocampal neurons

All use of animals was approved by the institutional animal care and use committee of the University of Copenhagen. All experiments were performed in accordance with the relevant guidelines and regulations. Primary cultures of rat hippocampal neurons were obtained as described previously^61^. In summary, whole brains were removed from E18/E19 rat embryos. Hippocampi were dissected, and the cells dissociated and cultured on poly-L-lysine treated coverslips that were placed upon an astrocyte feeder layer. Hippocampal neurons were cultured 10–15 days *in vitro* and afterwards analyzed with the patch–clamp technique.

### Electrophysiology

Whole-cell ionic currents were recorded as previously described^62^. In summary, whole-cell current recordings were performed at room temperature (20–22 °C) utilizing an Axopatch-200B/700B amplifier (Axon instruments, Union City, CA, USA), sampled at a 1–10 kHz frequency using a Digidata 1440/1550 acquisition system (Axon instruments) and filtered with a low-pass Bessel filter. The pClamp10 software (Axon instruments) controlled the command voltages and managed the data storage. HEK293 cells were continuously perfused with extracellular solution (ECS) containing (in mM): 145 NaCl, 4 KCl, 1 MgCl_2_, 1 CaCl_2_, 10 HEPES and 10 Glucose, adjusted to a pH of 7.35 with NaOH. Patch pipettes (1.5–2.5 MΩ) were pulled from borosilicate glass capillaries, heat polished and filled with an intracellular solution (ICS) containing (in mM): 110 KCl, 5 K_2_ATP, 5 K_4_BAPTA, 2 MgCl_2_ and 10 HEPES, pH adjusted to 7.2 with KOH. ICS and ECS solutions were used to record in HEK293 cells as well as in native hippocampal neurons. Where mentioned, 5 μM TTX was added to the ECS solution to inhibit native Na^+^ currents. When leak currents exceeded 10% of the total ionic current or voltage errors at the highest used potential exceeded the cut-off value of 5 mV (after series resistance compensation), cells were excluded from analysis.

### Drug solutions

All drug solutions were applied to the HEK293 cells using a fast perfusion system (ALA scientific Instruments, Farmingdale, NY, USA). Control recordings were obtained with fast perfusion of ECS solution. Retigabine (Alomone Labs, Jerusalem, Israel) was dissolved in DMSO to obtain a stock solution of 100 mM. The working concentrations (0.1–300 μM) were obtained by diluting the stock solution in ECS solution. Guangxitoxin–1E (Alomone Labs) and TTX (Sigma-Aldrich, Schnelldorf, Germany) were dissolved and diluted in ECS solution to obtain the stock (100 μM and 5 mM) and working (100 nM and 5 μM) solutions.

### Data analysis and statistics

The Hill equation: 1 − y = 1/(1 + (EC_50_/[D])^n^_H_), was fitted to concentration-effect curves to obtain a relative measurement of drug affinity with EC_50_ the concentration that induces 50% of the effect and n_H_ the Hill coefficient. For Kv7 channels the effect is defined as the induced shift in the voltage–dependence of activation normalized to the maximal shift (i.e. ΔV/ΔV_max_) while for Kv2.1 the degree of current inhibition (%) was plotted. The Boltzmann equation: y = 1/(1 + exp (−(V − V_1/2_)/k)), was applied to fit the voltage–dependence of (in)activation, where V represents the applied potential, V_1/2_ the voltage where 50% of the channels are (in)activated, and k the slope factor. Results were reported as the mean value ± S.E.M. Standard t–test or the Mann–Whitney Rank Sum test were used to determine whether the results achieved statistical significance. Statistical significance was defined as P < 0.05. Pulse protocols were adjusted to match the biophysical properties of the respective Kv channel and are illustrated throughout the figures.

## Additional Information

**How to cite this article**: Stas, J. I. *et al*. The anticonvulsant retigabine suppresses neuronal K_V_ 2-mediated currents. *Sci. Rep.* **6**, 35080; doi: 10.1038/srep35080 (2016).

## Supplementary Material

Supplementary Information

Supplementary Figure 1

Supplementary Figure 2

Supplementary Figure 3

## Figures and Tables

**Table 1:** Biophysical properties of K_V_2.1.

	Activation			Inactivation		
	V_1/2_	k	n	V_1/2_	k	n
K_V_2.1	3.7 ± 1.5	8.7 ± 0.4	12	−21.8 ± 1.0	6.2 ± 0.3	7
100 μM RTG	−2.5 ± 1.6	10.0 ± 1.4	9	−24.2 ± 1.6	5.9 ± 0.4	10
**RTG incubation**						
Control	3.8 ± 1.0	9.0 ± 0.5	14	−21.4 ± 2.6	5.4 ± 0.2	5
0.1% DMSO	1.9 ± 1.8	8.3 ± 0.3	11	−22.8 ± 3.0	5.60.2	5
0.1 μM RTG	4.9 ± 1.0	8.8 ± 0.3	12			
0.3 μM RTG	3.9 ± 1.5	8.8 ± 0.5	13			
1 μM RTG	6.3 ± 1.6	8.9 ± 0.6	12	−17.0 ± 1.5	5.8 ± 0.4	5
3 μM RTG	6.7 ± 1.2	8.0 ± 0.4	12			
**Inhibition Kinetics**						
	**τ**_**wash-in**_ **(s)**	**τ**_**wash-out**_ **(s)**		**% Inhibition**	**% Recovery**	**n**
100 μM RTG	89.7 ± 14.4	574 ± 67		80.8 ± 2.7	41.8 ± 7.6	8

V_1/2_, midpoint of activation or inactivation; k, slope factor; n, number of cells; τ_wash-in_, the rate of inhibition; τ_wash-out_, the rate of recovery. Values significantly different from the control values are shown in bold (p < 0.05).
