# Supplementary material for: The anticonvulsant retigabine suppresses neuronal KV2-mediated currents
Source: Sci Rep. 2016 Oct 13;6:35080. doi: 10.1038/srep35080 (PMC5062084; doi:10.1038/srep35080)
Supplement: Supplementary Information [file srep35080-s1.pdf]

Supplementary Information

The anticonvulsant retigabine suppresses neuronal K_V2-mediated currents.

Jeroen I. Stas[1,2], Elke Bocksteins[1], Camilla S. Jensen[2], Nicole Schmitt[2], Dirk J. Snyders[1*]

[1]Laboratory for Molecular Biophysics, Physiology and Pharmacology, Department of Biomedical Sciences, University of Antwerp, CDE, Universiteitsplein 1, 2610 Antwerp, Belgium

[2]Ion Channel Group, Department of Biomedical Sciences, University of Copenhagen, Blegdamsvej 3, DK-2200 Copenhagen N, Denmark

<u>Corresponding author:</u>

* Dirk J. Snyders, Laboratory for Molecular Biophysics, Physiology and Pharmacology, Department of Biomedical Sciences, University of Antwerp, CDE, Universiteitsplein 1, 2610 Antwerp, Belgium

E-mail: dirk.snyders@uantwerpen.be

Phone: +32-(3)-265-2335

Fax: +32-(3)-265-2326

Supplementary Figures

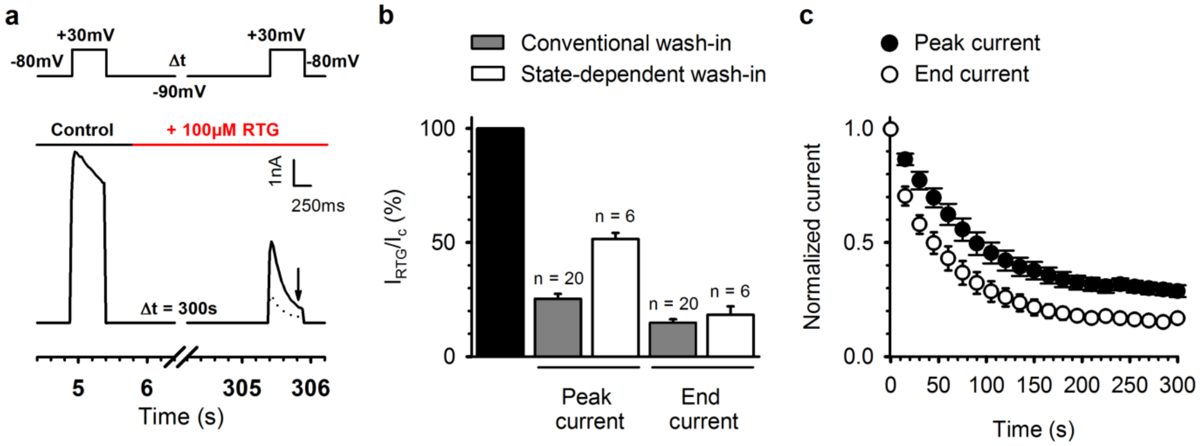

Supplementary Figure S1: State-dependence of K_V2.1 inhibition by retigabine. (a) Test for closed-state inhibition by retigabine in K_V2.1 channels. The voltage protocol is shown on top, and consisted of a twin pulse protocol to +30 mV, separated by a 300 s interval to -90 mV where all channels were closed and saturated inhibition should occur (see Figure 4). The first test pulse served as control. After the cells were exposed to 100 µM retigabine, the second test pulse provided a direct readout for potential closed-state inhibition. Although retigabine caused an instantaneous inhibition of the K_V2.1 current, inhibition was significantly different from the saturated inhibition (dotted line) which was determined by subsequent recording of a train of pulses. Note the large time-dependent decline of K_V2.1 current during this first step in the presence of retigabine. (b) Comparison of the degree of inhibition between the conventional (grey bars) and state-dependent (white bars) application of 100 µM retigabine. The remaining current (I/I_c) was determined (%) for both the peak current and then 'end' current (indicated with arrow in panel a) of the test pulse to +30 mV. (c) Normalized 'peak' (filled circles) and 'end' (open circles) currents of current traces during a 'conventional' wash-in experiment. The recorded 'peak' currents were always higher than the 'end' current of the previous current trace suggesting either partial recovery of inhibition or from slow inactivation during the interval between recording of the traces (i.e. 15 s at -80 mV). However, clearly no additional inhibition developed at -80 mV.

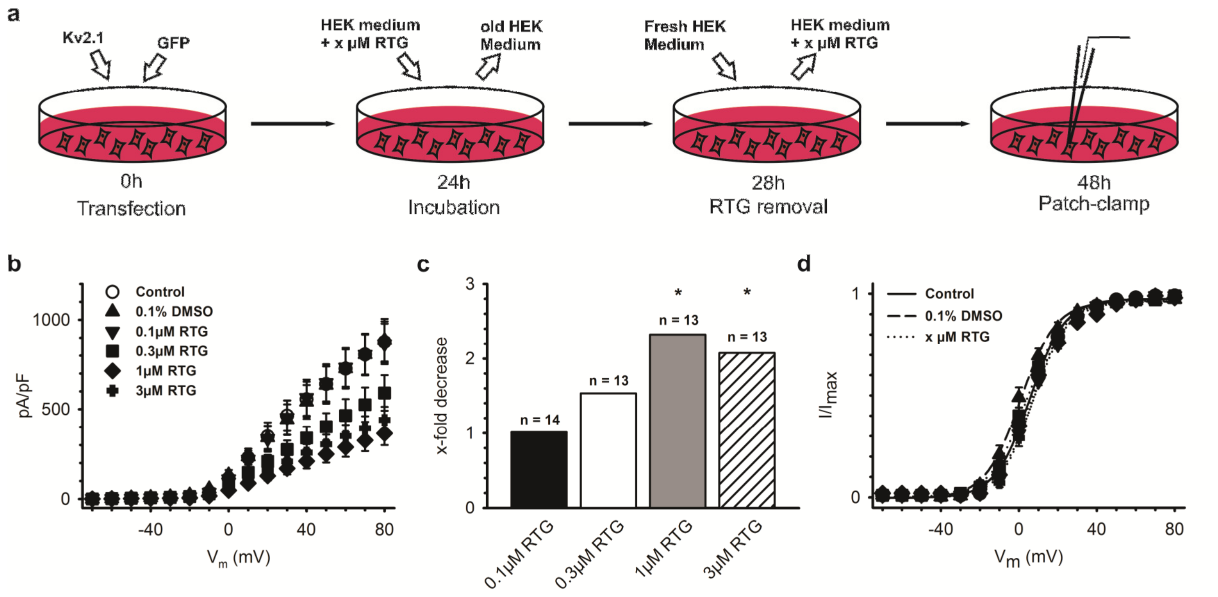

Supplementary Figure S2: Retigabine modulates $K_V2.1$ current density upon prolonged exposure. (**a**) Procedure to study the effect of prolonged exposure of low retigabine concentrations on $K_V2.1$ currents. Detailed description is provided in the material & methods section. (**b**) $K_V2.1$ current densities under control conditions (open circles) and after incubation with 0.1% DMSO (▲) and increasing retigabine concentrations, 0.1 µM (▼), 0.3 µM (■), 1 µM (♦) and 3 µM (+). Retigabine caused a concentration-dependent decrease of the $K_V2.1$ current densities. (**c**) Fractional reduction in $K_V2.1$ current densities as a function of the retigabine concentration. * indicates statistical significance ($p < 0.05$). (**d**) Voltage-dependence of activation. No change in the voltage-dependence of activation occurred as a consequence of the manipulation or exposure to either vehicle or retigabine concentrations. Lines represent fit of the voltage-dependence of activation with the Boltzmann equation.

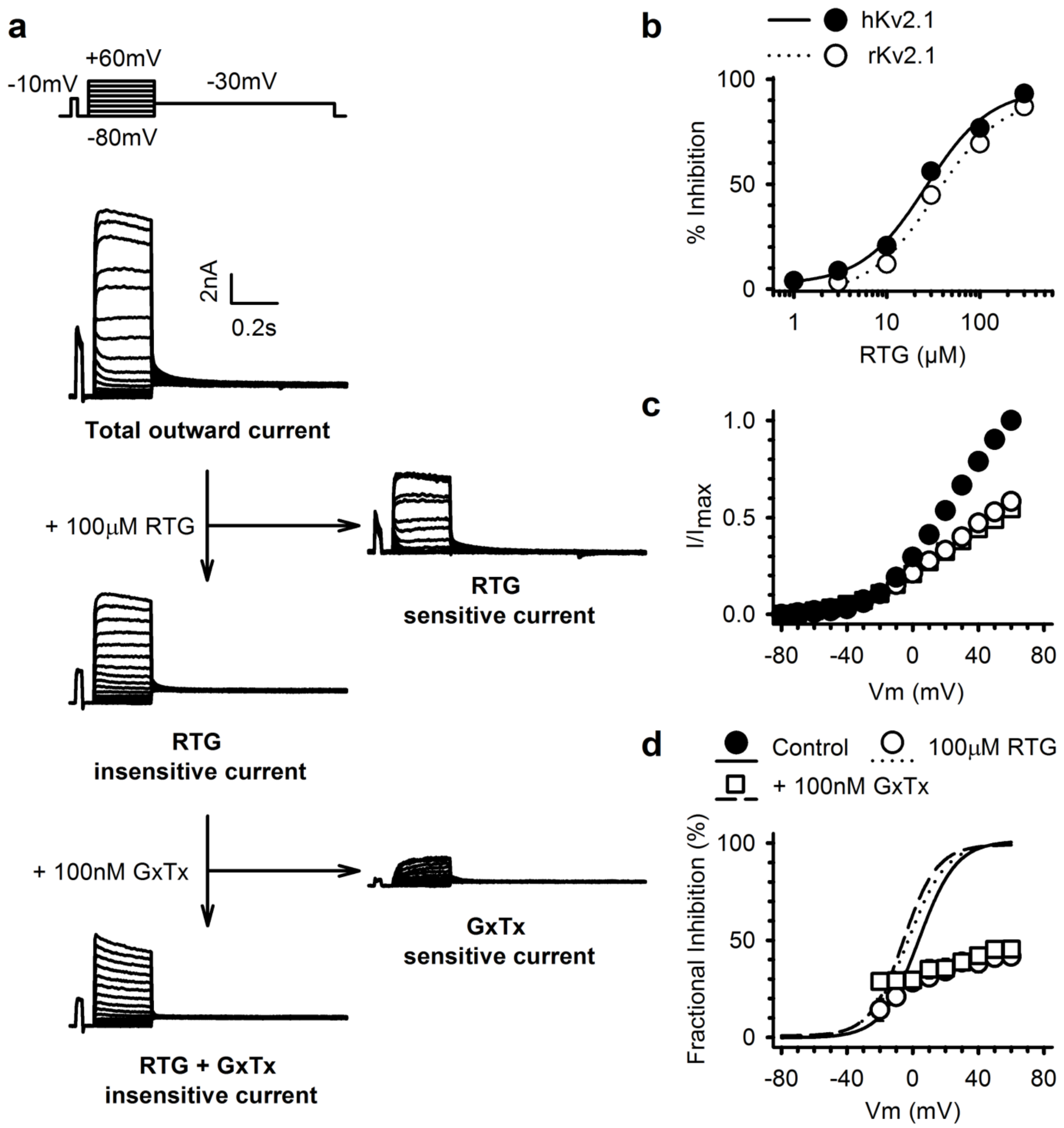

Supplementary Figure S3: Retigabine inhibits the K_V2-mediated component of the outward current of rat hippocampal neurons in the presence of TTX. (**a**) Representative current traces from cultured rat hippocampal neurons with 5 µM TTX in the bathing solution. The outward current was inhibited with retigabine and GxTx as previously described in Fig. 5. Retigabine inhibited the K_V2-mediated component of the outward current, with little additional inhibition caused by GxTx. (**b**) Concentration-effect relationship of human K_V2.1 (filled circles) and rat K_V2.1 (open circles) current inhibition. Rat K_V2.1 was slightly less sensitive which might underscore the effect of retigabine on the outward currents in Fig. 5. (**c**) Current-voltage relationship obtained as described in Fig. 5c, d. (**d**) Fractional inhibition of retigabine (open circles) and subsequent exposure to GxTx (grey circles). Retigabine had a strong voltage-dependence of inhibition. Lines represent fit of the voltage-dependence of activation with the Boltzmann equation.

Supplementary Tables

Supplementary Table S1: Activation properties of $K_V7.2$-$K_V7.3$ channels (± KCNE2).

	$K_V7.2$-$K_V7.3$				$K_V7.2$-$K_V7.3$ + KCNE2			
	$V_{1/2}$	k	n	$\Delta V/\Delta V_{max}$ *	$V_{1/2}$	k	n	$\Delta V/\Delta V_{max}$ **
**Control**	-16.3 ± 1.5	9.3 ± 0.2	43		-19.6 ± 1.2	8.9 ± 0.3	22	
**Retigabine (µM)**								
**1**	-27.1 ± 2.5	9.5 ± 0.4	14	27.1 ± 3.2	-21.0 ± 2.3	8.6 ± 0.3	8	3.0 ± 3.8
**3**	-41.0 ± 2.2	9.0 ± 0.3	16	56.1 ± 5.3	-26.7 ± 1.9	9.1 ± 0.4	12	25.4 ± 4.9
**10**	-46.7 ± 2.3	9.2 ± 0.4	14	77.9 ± 4.2	-36.0 ± 1.9	9.0 ± 0.2	11	54.0 ± 6.4
**30**	-54.0 ± 0.9	8.8 ± 0.3	14	88.7 ± 5.3	-45.9 ± 1.5	8.4 ± 0.2	8	86.1 ± 6.7
**100**	-55.5 ± 2.1	8.6 ± 0.3	11	95.2 ± 4.4	-49.2 ± 1.6	8.6 ± 0.4	9	98.3 ± 3.5
**300**	-55.0 ± 1.2	9.3 ± 0.4	14	99.9 ± 3.5	-49.0 ± 2.8	8.8 ± 1.1	9	97.8 ± 4.8
**EC_{50}**		1.9 ± 1.5				10.0 ± 2.2		
**ΔV_{max} (mV)**		39.2 ± 1.4				30.7 ± 1.1		

$V_{1/2}$, midpoint of activation; k, slope factor; n, number of cells; ΔV, the shift in the voltage-dependence of activation; ΔV_{max}, the maximal shift in the voltage-dependence of activation. Values significantly different from the control values are shown in bold ($p < 0.05$). * ΔV_{max} of $K_V7.2$-$K_V7.3$ channels.** ΔV_{max} of $K_V7.2$-$K_V7.3$ + KCNE2 channels
