# Supplementary figures and images for: The anticonvulsant retigabine suppresses neuronal KV2-mediated currents
Source: Sci Rep. 2016 Oct 13;6:35080. doi: 10.1038/srep35080 (PMC5062084; doi:10.1038/srep35080)

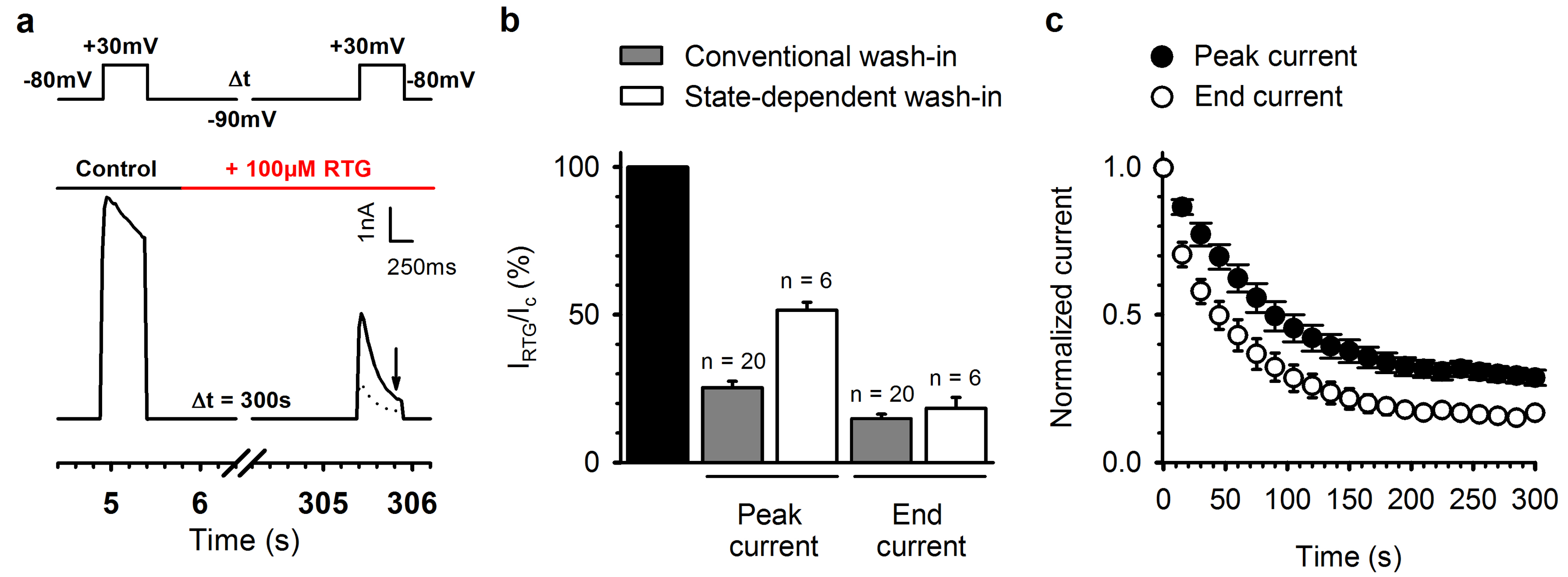

Supplement: Supplementary Figure 1 [file srep35080-s2.jpg]

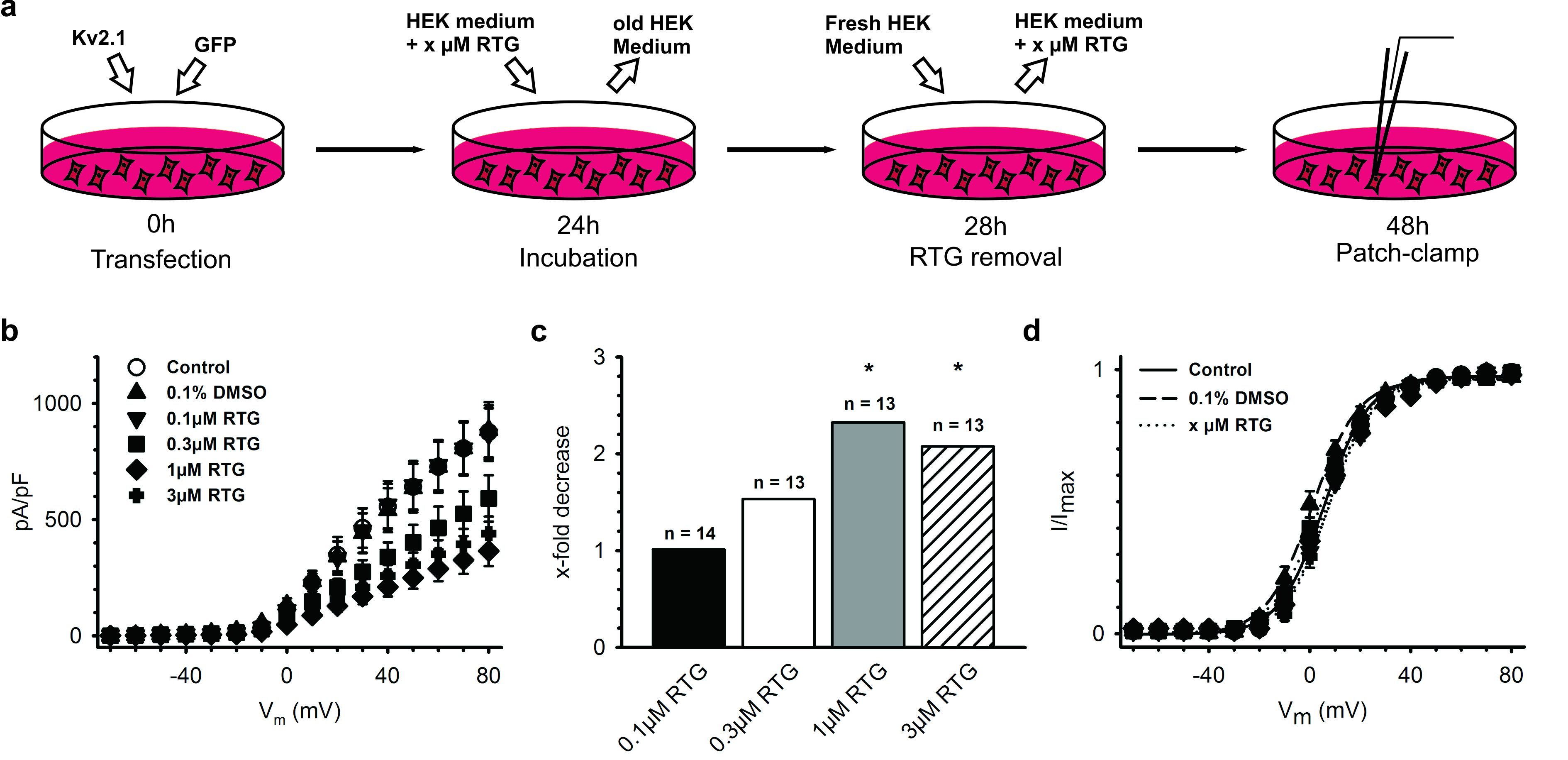

Supplement: Supplementary Figure 2 [file srep35080-s3.jpg]

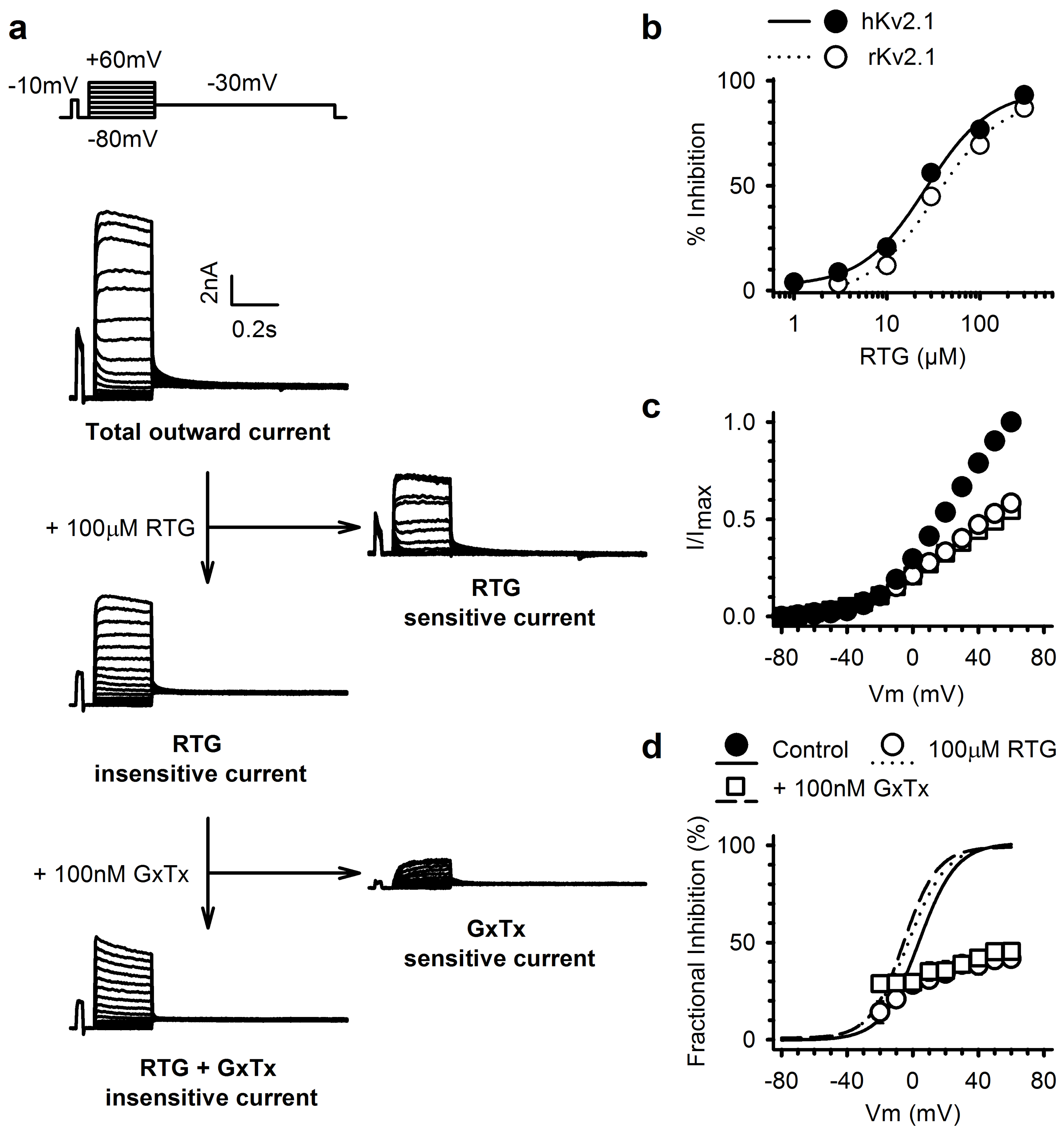

Supplement: Supplementary Figure 3 [file srep35080-s4.jpg]
